# Effects of Annealing and Thickness of Co_60_Fe_20_Yb_20_ Nanofilms on Their Structure, Magnetic Properties, Electrical Efficiency, and Nanomechanical Characteristics

**DOI:** 10.3390/ma15155184

**Published:** 2022-07-26

**Authors:** Wen-Jen Liu, Yung-Huang Chang, Yuan-Tsung Chen, Po-Chun Chiu, Jian-Cheng Guo, Shih-Hung Lin, Po-Wei Chi

**Affiliations:** 1 Department of Materials Science and Engineering, I-Shou University, Kaohsiung 84001, Taiwan; jurgen@isu.edu.tw; 2 Bachelor Program in Interdisciplinary Studies, National Yunlin University of Science and Technology, 123 University Road, Section 3, Douliou 64002, Yunlin County, Taiwan; changyhu@yuntech.edu.tw; 3 Graduate School of Materials Science, National Yunlin University of Science and Technology, 123 University Road, Section 3, Douliou 64002, Yunlin County, Taiwan; m10947005@yuntech.edu.tw (P.-C.C.); m11047006@yuntech.edu.tw (J.-C.G.); 4 Department of Electronic Engineering, National Yunlin University of Science and Technology, 123 University Road, Section 3, Douliou 64002, Yunlin County, Taiwan; isshokenmei@yuntech.edu.tw; 5 Institute of Physics, Academia Sinica, Nankang, Taipei 11529, Taiwan; jacky01234567891@hotmail.com

**Keywords:** annealed Co_60_Fe_20_Yb_20_ thin films, X-ray diffraction (XRD), low-frequency alternating current magnetic susceptibility (χ_ac_), adhesion, electrical properties, nanomechanical properties

## Abstract

X-ray diffraction (XRD) analysis showed that metal oxide peaks appear at 2θ = 47.7°, 54.5°, and 56.3°, corresponding to Yb_2_O_3_ (440), Co_2_O_3_ (422), and Co_2_O_3_ (511). It was found that oxide formation plays an important role in magnetic, electrical, and surface energy. For magnetic and electrical measurements, the highest alternating current magnetic susceptibility (χ_ac_) and the lowest resistivity (×10^−2^ Ω·cm) were 0.213 and 0.42, respectively, and at 50 nm, it annealed at 300 °C due to weak oxide formation. For mechanical measurement, the highest value of hardness was 15.93 GPa at 200 °C in a 50 nm thick film. When the thickness increased from 10 to 50 nm, the hardness and Young’s modulus of the Co_60_Fe_20_Yb_20_ film also showed a saturation trend. After annealing at 300 °C, Co_60_Fe_20_Yb_20_ films of 40 nm thickness showed the highest surface energy. Higher surface energy indicated stronger adhesion, allowing for the formation of multilayer thin films. The optimal condition was found to be 50 nm with annealing at 300 °C due to high χ_ac_, strong adhesion, high nano-mechanical properties, and low resistivity.

## 1. Introduction

The direction of information storage has gradually moved toward magnetic recording. CoFe alloys have good mechanical properties and soft magnetic characteristics, and they are the most commonly studied alloys currently due to high saturation magnetization (M_S_) and low coercivity (H_c_) [1,2,3,4,5,6]. Due to its excellent characteristics, the Co-Fe system has received extensive attention. At present, Co-Fe alloys are often used in magnetic devices, sensors, actuators, and magnetic storage [7,8,9,10,11,12,13,14,15]. Magnetic properties are affected by thickness, crystallinity, and interfacial interactions. Annealing is usually needed to improve the tunneling magnetoresistance (TMR) ratio of CoFeB/MgO structures. When the crystallization temperature is higher than the temperature of the sample, the nanocrystal structure exhibits good soft magnetic efficiency, which is related to the strong inter-particle magnetic exchange mediated by the amorphous ferromagnetic matrix. When the amorphous state changes to a nanocrystalline structure, the TMR is changed [16,17,18]. However, adding a third rare earth element to CoFe alloy materials, such as La, Ce, Sm, Gd, Dy, Ho, Er, or Yb, may be one of the ways to break through the current predicament. Rare earth elements have unique properties that can be used to improve high temperature resistance, such as the mechanical strength and other physical properties of magnetic films. Perhaps rare earth elements can be used to improve and resolve the thermal stability of magnetic films at high temperatures. Adding Yb was found to improve crystallinity. Generally, a higher degree of crystallinity signifies more molecular chain arrangement, which requires a higher temperature to destroy, and the melting point is also increased. This also indirectly shows that adding Yb can improve the heat resistance of the material [19,20,21,22,23,24]. Moreover, a Co_60_Fe_20_B_20_ thin film can be commonly used as a free or pinned layer in a magnetic tunneling junction (MTJ) structure, and it is necessary to improve TMR and thermal stability. The main motivation of this research is that Co_60_Fe_20_Yb_20_ is a new material in the field of magnetism that will replace Co_60_Fe_20_B_20_ films in MTJ structures as a free or pinned layer to improve magnetic properties and thermal stability. However, the disadvantages of CoFe films include brittleness and reduced magnetic characteristics at high temperatures. The mechanical strength and magnetic properties of CoFe films can be improved by adding Yb. The addition of Yb as the third element may improve the mechanical properties of CoFe alloys. The purpose of this research is to study the structure and magnetic properties of CoFeYb thin films as a function of thickness, and to study annealed CoFeYb thin films to determine whether they will change due to high-temperature environments. The performance of CoFeYb as a free or pinned layer of an MTJ device is sensitive to a higher temperature environment. In addition, its magnetic anisotropy could be seriously degraded in a CoFeB-MgO system when the annealing temperature is more than 350 °C [25]. Accordingly, it is worthwhile to study the specific properties and thermal stability of CoFeYb films at annealed temperatures from 100 to 300 °C. This study mainly measured the structure, magnetic properties, electrical properties, mechanical properties, contact angle, and adhesion efficiency of Co_60_Fe_20_Yb_20_ thin films with various thicknesses and annealing temperatures. The X-ray diffraction results indicate that the oxidation of the film at each thickness was roughly the same, and the proportion of oxides increased in thinner films. It was found that the oxide formation plays an important role in magnetic, electrical, and surface energy.

## 2. Materials and Methods

A 10–50 nm CoFeYb thin film was deposited on a Si(100) substrate by direct-current (DC) magnetron sputtering. The film was prepared under the following four conditions: (a) at room temperature (RT), (b) annealing at 100 °C for 1 h, (c) annealing at 200 °C for 1 h, and annealing at 300 °C 1 h. The base pressure of the sputtering vacuum was 3.5 × 10^−7^ Torr, and the Ar working pressure was 3 × 10^−3^ Torr. The pressure in the annealed condition was 2 × 10^−3^ Torr. The alloy target for the composition of CoFeYb was 60 at% Co, 20 at% Fe, and 20 at% Yb. In addition, the structure of the CoFeYb films was verified using the oblique incident X-ray diffraction (GIXRD) pattern obtained by CuKα1 (PAN Analytical X`pert PRO MRD). For the magnetic properties, the low-frequency alternate-current magnetic susceptibility (χ_ac_) of the Co_60_Fe_20_Yb_20_ films was investigated using a χ_ac_ analyzer (XacQuan). For electrical properties, resistivity and sheet resistance (Rs) were studied by using a conventional four-point technique. The measurement methods can be divided into two types: fixed voltage or fixed current. Both methods conform to Ohm’s law R = V/I. The hardness and Young’s modulus of the Co_60_Fe_20_Yb_20_ films were investigated using the MTS Nano Indenter XP with a Berkovich tip and continuous stiffness measurement (CSM) technique. Then, the indent was withdrawn from the surface at the same rate until the load was reduced to 10% of the maximum load. The measurement was repeated with the probe at 10 positions for each sample. The indentation load was added in 40 steps, and the indentation depth was measured in each step. Six indentations were examined in each sample, and the standard deviations were averaged to provide more accurate results. The contact angles were measured using deionized (DI) water and glycerol. Finally, the surface energy was calculated according to the contact angle [26,27,28].

## 3. Results

### 3.1. X-ray Diffraction and Full Width at Half Maximum (FWHM)

**Figure 1:**
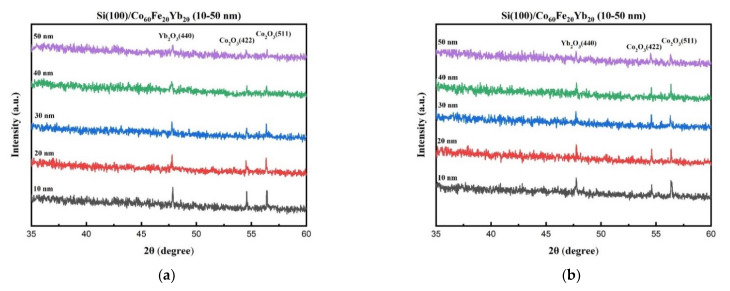

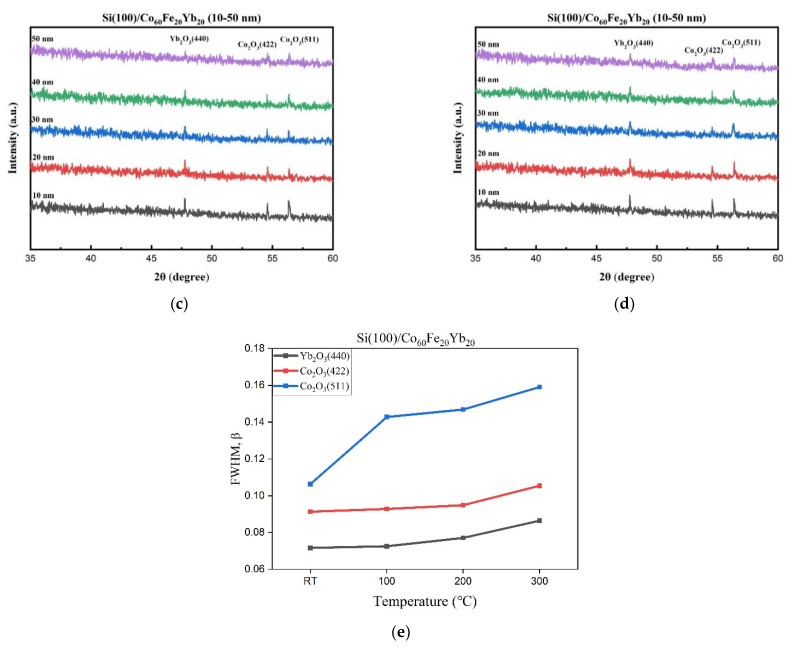
XRD patterns of CoFeYb films. (**a**) RT, (**b**) after annealing at 100 °C, (**c**) after annealing at 200 °C, (**d**) after annealing at 300 °C. (**e**) FWHM of oxide peaks in CoFeYb 40 nm at various conditions.

Figure 1a–d shows the X-Ray diffraction (XRD) patterns of the CoFeYb thin films as deposited and annealed with thicknesses from 10 to 50 nm. Figure 1a shows the pattern of the as-deposited nanofilms, while the models of the annealed nanofilms at 100, 200 and 300 °C are shown in Figure 1b–d. Figure 1a–d shows that the oxide peaks appear at 2θ = 47.7°, 54.5°, and 56.3° in all CoFeYb samples. They correspond to Yb_2_O_3_ (440), Co_2_O_3_ (422), and Co_2_O_3_ (511). The chamber of the sputtering system is pumped up to 10^−7^ Torr, but oxygen may still be present. Both natural oxides on the Si (100) substrate and oxygen contamination on the sputtering target contribute to the formation of oxidation peaks [29]. Figure 1a–d shows that as the thickness of CoFeYb increases, the intensities of all oxide peaks decrease. The oxidation of the film at each thickness was roughly the same; thus, when the film thickness was thinner, the proportion of oxides increased. Hence, the intensity of the oxide peaks gradually weakened as the thickness increased. The weakening peak of the oxide can reduce its interference and improve the magnetism and electrical properties of the film. Moreover, XRD also shows no apparent diffracted peak in the CoFeYb films, indicating that the films are amorphous even at higher annealing temperatures. It can be concluded reasonably that the thermal driving force is not enough to support grain growth. Figure 1e shows the full width at half maximum (FWHM) of oxide peaks in CoFeYb 40 nm at various conditions. From this result, the FWHM of annealed CoFeYb films is larger than the as-deposited film, which indicates that the structural quality of the annealed films is weaker than the as-deposited films.

### 3.2. Magnetic Property

**Figure 2:**
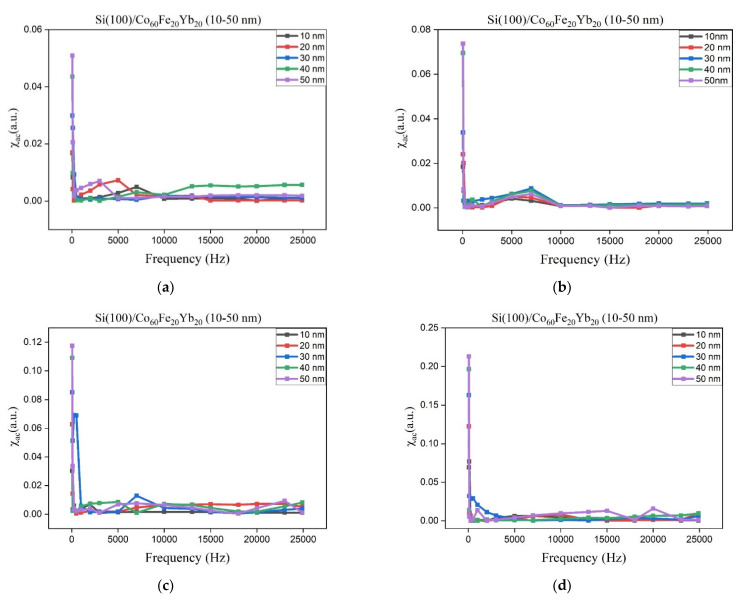
χ_ac_ as a function of frequency from 50 to 25,000 Hz. (**a**) RT, (**b**) after annealing at 100 °C, (**c**) after annealing at 200 °C, (**d**) after annealing at 300 °C.

Figure 2a–d shows that χ_ac_ has a thickness of 10 to 50 nm at room temperature as well as at 100, 200 and 300 °C. In the low frequency range of 50–25,000 Hz, χ_ac_ decreases as the frequency increases. The results also show that when the thickness is from 10 to 50 nm, χ_ac_ is increased.

**Figure 3:**
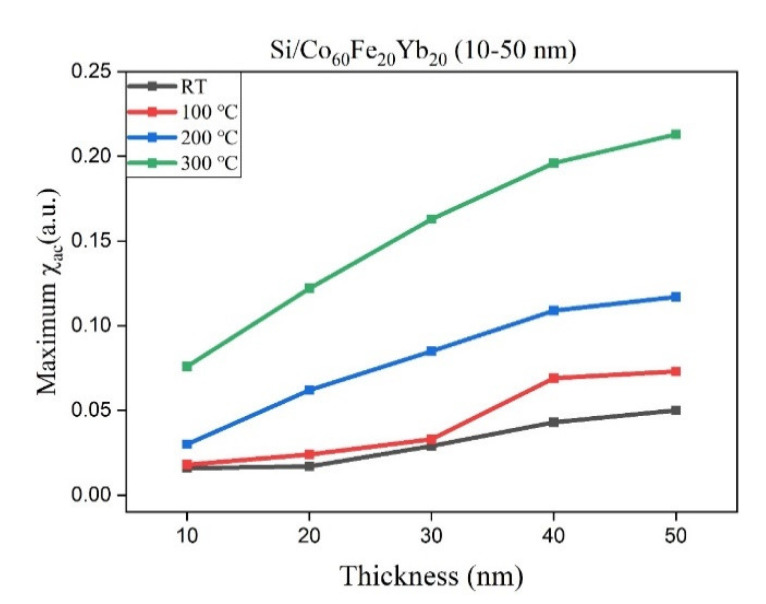
Maximum χ_ac_ for the CoFeYb films.

Under the four preparation conditions, the corresponding maximum χ_ac_ under different CoFeYb thicknesses is shown in Figure 3. The peak value of χ_ac_ was at 300 °C and annealed at 50 nm, which is higher than the value under other conditions in this study. The results show that the χ_ac_ trend is consistent with the XRD results: as the thickness of CoFeYb increases, the intensities of all oxide peaks decrease, which also exhibits a tendency to increase magnetism. It can be reasonably concluded that antiferromagnetic oxidation is formed in the CoFeYb film. The results clearly show the effect of χ_ac_ thickness on all CoFeYb samples. As the thickness of CoFeYb increases, the maximum χ_ac_ value increases due to the thickness effect. The maximum χ_ac_ value of the CoFeYb thin film after annealing was larger than the maximum χ_ac_ value of the as-deposited sample. Table 1 lists the maximum χ_ac_ corresponding to the optimal resonant frequency (f_res_) under the four conditions. At f_res_, the maximum χ_ac_ has the strongest spin sensitivity [30,31]. The f_res_ value of CoFeYb at various thicknesses in this study was less than 250 Hz, which can be used in low-frequency magnetic applications.

### 3.3. Electrical Properties

**Figure 4:**
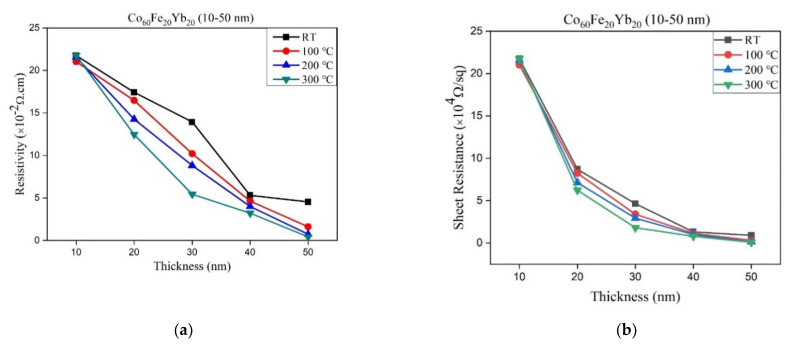
(**a**) The resistivity of CoFeYb films and (**b**) the sheet resistance of CoFeYb films.

The resistivity and sheet resistance (Rs) are shown in Figure 4a,b with different thicknesses and annealed temperatures. The results of this study show that both resistivity and sheet resistance decrease as the thickness and annealing temperature increase. It is speculated that oxides at higher annealing temperatures can reduce the resistance of the current in the flow and reduce the resistivity [32,33]. According to the XRD results and Figure 1e, it can be found that the oxidation of the film at each thickness was roughly the same, and the proportion of oxides increased with thinner thicknesses. Hence, the intensity oxides peaks gradually weakened in the thicker films, and reduced oxide causes lower resistivity to electron transport in the CoFeYb film. It can be concluded that the oxide formation hinders electron carrier conduction and increases resistivity.

### 3.4. Nano-Indentation

Table 2 and Table 3 show that the hardness and Young’s modulus increase as the thicknesses increase from 10 to 50 nm. Generally, the hardness is determined from the loading and unloading curves by the Pharr–Oliver method [34], which indicates the mixed hardness of the silicon substrate and CoFeYb films. Since the CoFeYb film is thin, it is reasonable to assume that there must be a substrate effect in the nano-indentation measurement. The results in Table 2 and Table 3 indicate that as the thickness increases from 10 to 50 nm, the hardness and Young’s modulus increase from 10.2 to 15.16 GPa and from 190 to 229.13 GPa at RT. At the annealing temperature of 100 °C, the hardness and Young’s modulus increase from 9.37 to 15.74 GPa and from 195.96 to 231.08 GPa with an increase in thickness from 10 to 50 nm. At an annealing temperature of 200 °C, as the thickness increases from 10 to 50 nm, the hardness and Young’s modulus increase from 9.91 to 15.93 GPa and from 214.97 to 234.85 GPa, respectively. At an annealing temperature of 300 °C, as the thickness increases from 10 to 50 nm, hardness and Young’s modulus increase from 10.26 to 15.34 GPa and from 216.42 to 230.78 GPa, respectively. The experimental results demonstrate that although temperature does not significantly change the hardness and modulus, the hardness and Young’s modulus of Si(100) increase significantly as the film thickness increases, and the effect of thickness is significant [35,36].

### 3.5. Analysis of Surface Energy and Adhesion

**Figure 5:**
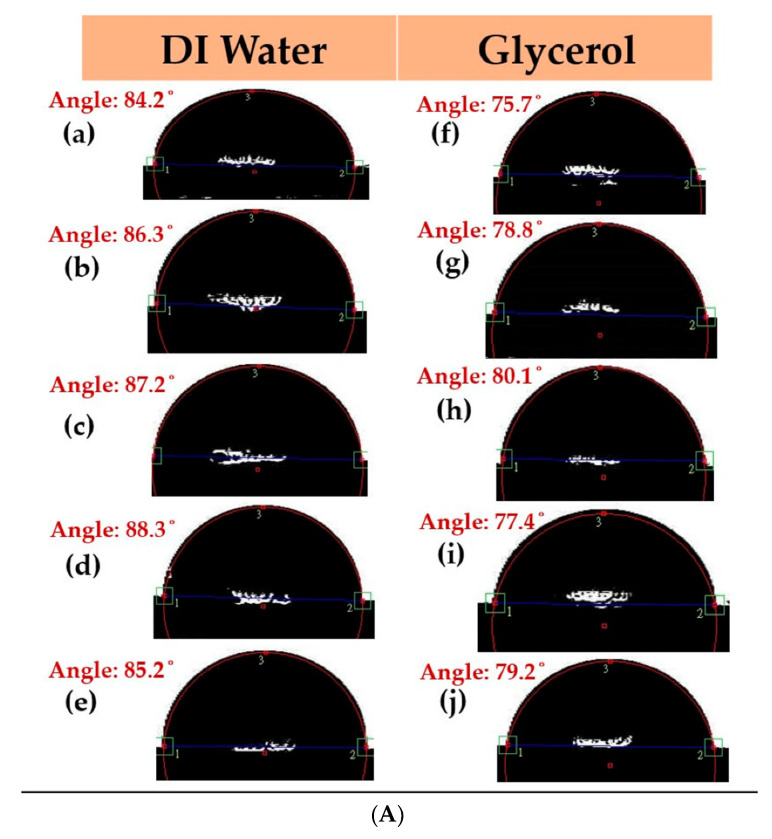

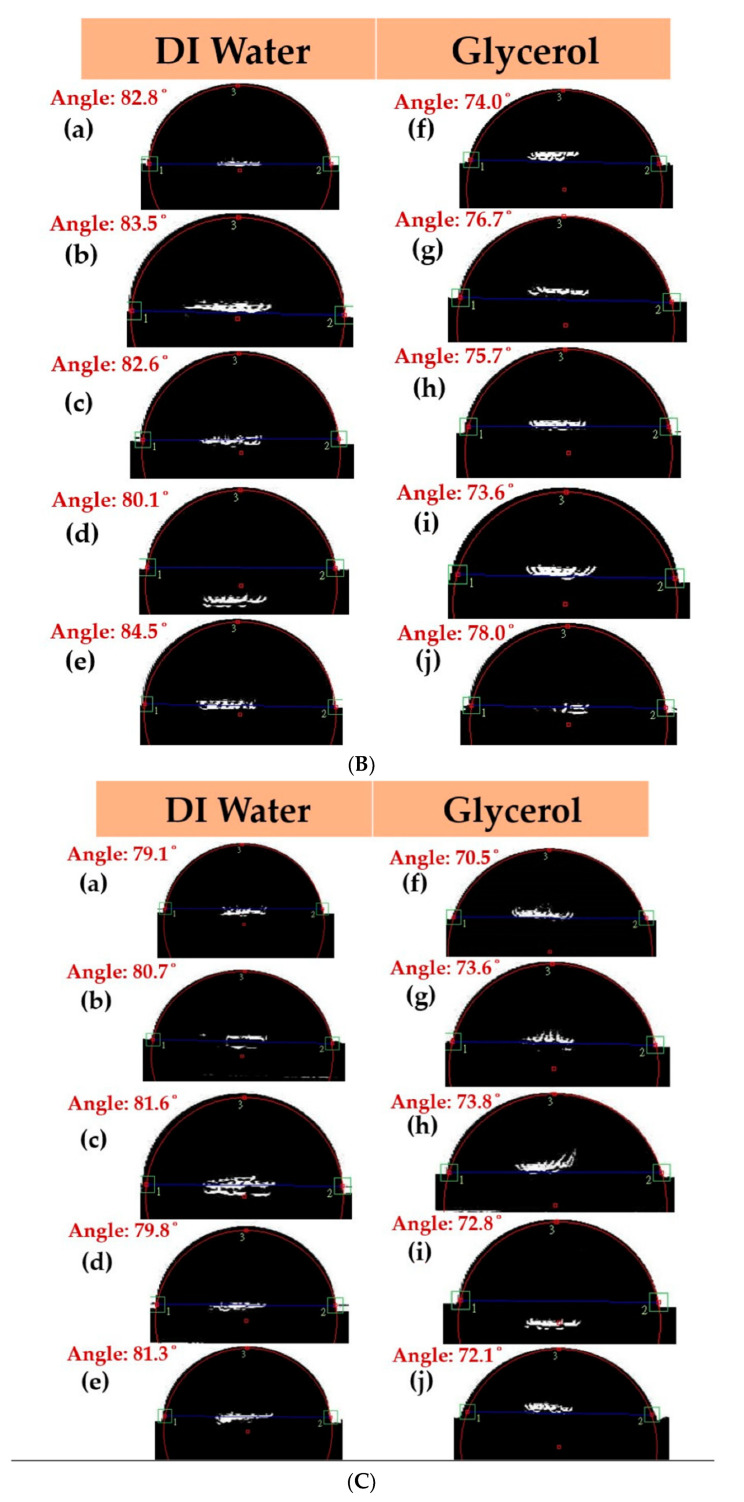

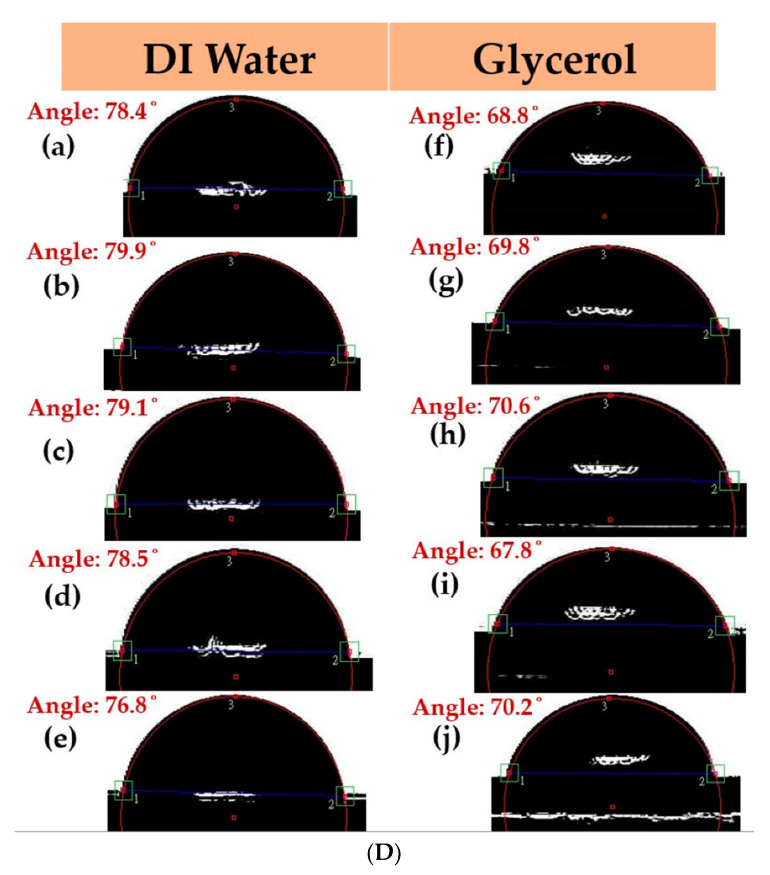
Contact angles of the CoFeYb thin films: (**A**) RT, (**B**) after annealing at 100 °C, (**C**) after annealing at 200 °C, (**D**) after annealing at 300 °C. DI water: (**a**) 10 nm, (**b**) 20 nm, (**c**) 30 nm, (**d**) 40 nm, and (**e**) 50 nm. Glycerol: (**f**) 10 nm, (**g**) 20 nm, (**h**) 30 nm, (**i**) 40 nm, and (**j**) 50 nm.

The contact angles were measured using DI water and glycerol, which is shown in Figure 5A–D. The contact angles under all conditions were observed to be less than 90°, and the droplets were almost spherical, indicating that the films have good hydrophilicity and wetting ability. Thus, it can be concluded that the contact angle tends to decrease as the annealing temperature increases. This result is mainly due to the increase in crystallization with annealing treatment. Surface energy and adhesion are important parameters, as CoFeYb films can be used as a seed or a buffer layer. When the surface energy is high, the absorption of the liquid is large and the contact angle is reduced. The adhesion efficiency and the contact angle were less than 90, suggesting the hydrophilic nature of the film on the Si(100) substrate.

**Figure 6:**
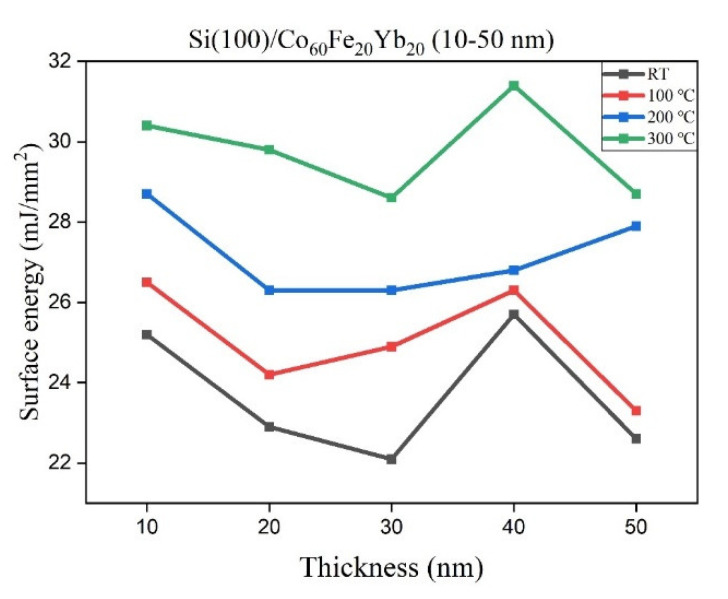
Surface energy of CoFeYb films.

Figure 6 shows the surface energy of the CoFeYb film, consisting of thicknesses that increase from 10 to 50 nm at RT and after annealing at 100, 200 and 300 °C. The results reveal that the surface energy is between 22.1 and 31.4 mJ/mm^2^, and the annealed film is higher than that of the film at RT. When the surface energy of the film is high, the adhesion is strongest. In Figure 6, there is behavior at a thickness of 40 nm for RT at 100 and 300 °C, which then decreases at 50 nm. However, when annealing at 200 °C, the behavior is different with respect to the other samples, as it is increasing. It can be reasonably assumed that the amount of oxides affects the surface energy [29,36]. According to the XRD results, the formation of more oxide layers on the thinner film surfaces results in increased contact angles and decreased surface energy [37].

To investigate the surface energy and maximum χ_ac_ of CoFeYb performance, Table 4 is compared with other specific CoFeBY and CoFeW materials under Si(100) substrate. Table 4 demonstrates that the surface energy of the current research is lower than other CoFeBY and CoFeW materials. Moreover, maximum χ_ac_ of the CoFeYb films is higher than that of the CoFeBY films.

## 4. Conclusions

The XRD patterns demonstrated that in all CoFeYb samples, the oxide peak appeared at 2 θ = 47.7°, 54.5° and 56.3°, corresponding to Yb_2_O_3_ (440), Co_2_O_3_ (422) and Co_2_O_3_ (511). When the film thickness was thinner, the proportion of oxides increased. Hence, the intensity of peaks of the oxides gradually weakened as the thickness increased. The weakening peak of the oxide can reduce its interference and improve the magnetism and electrical properties of the film. The magnetic properties also show this thickness effect. The electrical properties suggest that resistivity and sheet resistance both decreased as they increased in thickness and annealed temperatures due to fewer oxide formation. Nanomechanical properties indicate that the hardness and Young’s modulus increase with the increase in CoFeYb film thickness, and they create a substrate effect in nano-indentation measurement. The surface energy of the annealing CoFeYb films was higher than that of the as-deposited films. Based on the above results, the optimal condition was found to be 50 nm with annealing at 300 °C due to high χ_ac_, strong adhesion, high nano-mechanical properties, and low resistivity.

## Figures and Tables

**Table 1:** Optimal resonant frequency for films of various thicknesses.

Thickness	RT Optimal Resonant Frequency (Hz)	After Annealing at 100 °C Optimal Resonant Frequency (Hz)	After Annealing at 200 °C Optimal Resonant Frequency (Hz)	After Annealing at 300 °C Optimal Resonant Frequency (Hz)
10 nm	50	50	100	100
20 nm	250	50	50	50
30 nm	50	50	50	50
40 nm	50	50	50	50
50 nm	50	50	50	50

**Table 2:** Hardness of CoFeYb films.

Thickness (nm)	RT Hardness (GPa)	100 °C Hardness (GPa)	200 °C Hardness (GPa)	300 °C Hardness (GPa)
10	10.20	9.37	9.91	10.26
20	12.24	13.79	14.34	14.39
30	13.67	14.89	15.34	14.52
40	14.85	15.26	15.38	15.01
50	15.16	15.74	15.93	15.34

**Table 3:** Young’s modulus of CoFeYb films.

Thickness (nm)	RT Modulus (GPa)	100 °C Modulus (GPa)	200 °C Modulus (GPa)	300 °C Modulus (GPa)
10	190	195.96	214.97	216.42
20	216.97	209.23	231.07	227.26
30	217.41	224.27	232.43	227.78
40	220.58	227.30	232.86	228.18
50	229.13	231.08	234.85	230.78

**Table 4:** Comparing surface energy for specific CoFeW and CoFeBY thin films from Si(100) substrate.

	Surface Energy (mJ/mm^2^)	Maximum χ_ac_ (a.u.)
Si(100)/Co_60_Fe_20_Yb_20_ Current research	22.10–31.40	0.016–0.213
Si(100)/Co_40_Fe_40_W_20_ [36]	24.00–36.02	0.055–0.745
Si(100)/Co_40_Fe_40_B_10_Y_10_ [29]	24.55–31.85	0.022–0.185
Si(100)/Co_40_Fe_40_W_20_ [38]	23.61–30.12	0.02–0.18
